# Highlight: Sex As Stress Management in Microbes

**DOI:** 10.1093/gbe/evaa080

**Published:** 2020-04-22

**Authors:** Casey McGrath

Why is sex so popular? The question of why so many organisms reproduce sexually has mystified evolutionary biologists since before Darwin, who wrote that “The whole subject is as yet hidden in darkness.” In a recent article in *Genome Biology and Evolution* titled “What’s genetic variation got to do with it? Starvation-induced self-fertilization enhances survival in *Paramecium*,” the authors suggest that the molecular mechanisms underlying sex and the stress response may be more tightly coupled than previously appreciated, providing a new explanation for the widespread prevalence of sex in nature (Thind et al. 2020).

The existence of sex has puzzled biologists for over a century. Compared with asexual reproduction, sex has several disadvantages. The foremost of these is that each sexual organism produces only half as many offspring as asexual individuals. For example, if each adult has two children, two asexual individuals can produce four offspring, whereas two sexual individuals—one male and one female—produce only two offspring between them. From an evolutionary perspective, this is a staggering cost, even without taking into account other disadvantages of sex, such as the need to find a mate and the potential dangers of doing so (especially if you are a male praying mantis or black widow spider).

Despite these costs, sex is widespread, with an estimated 99% of eukaryotes (cells with nuclei) reproducing sexually at least some of the time. This paradox has resulted in a number of hypotheses attempting to explain the near-ubiquity of sex. According to Francesco Catania, lead author of the new study and a research group leader at the University of Münster, one popular explanation is that sex produces genetic diversity—this is why you and your siblings are not identical to your parents. The argument is that this genetic diversity may produce some individuals that are better adapted to changing or harsh environments. In contrast, asexual reproduction generally produces offspring that are each identical to the parent.

The single-celled ciliate *Paramecium tetraurelia* provides a fascinating counterpoint to this argument, as it can undergo both asexual reproduction and a version of sexual reproduction that notably does not produce genetic diversity (i.e., a kind of selfing). For most of their life cycle, paramecia reproduce asexually, with each cell splitting into two. When a cell reaches sexual maturity however, each paramecium may produce two identical sexual nuclei—similar to the nuclei that are present in sperm and egg cells. If another paramecium is not around to mate and exchange nuclei with, then these two nuclei fuse with each other. The result is a type of sexual self-fertilization that can result in daughter cells that are genetically identical to their parents. Thus, in *Paramecium*, sexual reproduction can be uncoupled from the generation of genetic diversity, suggesting that there are other potential benefits to sex in this organism. Catania and his coauthors realized that this makes *Paramecium* a unique model in which to investigate and potentially identify these other benefits.

**Fig. 1.**
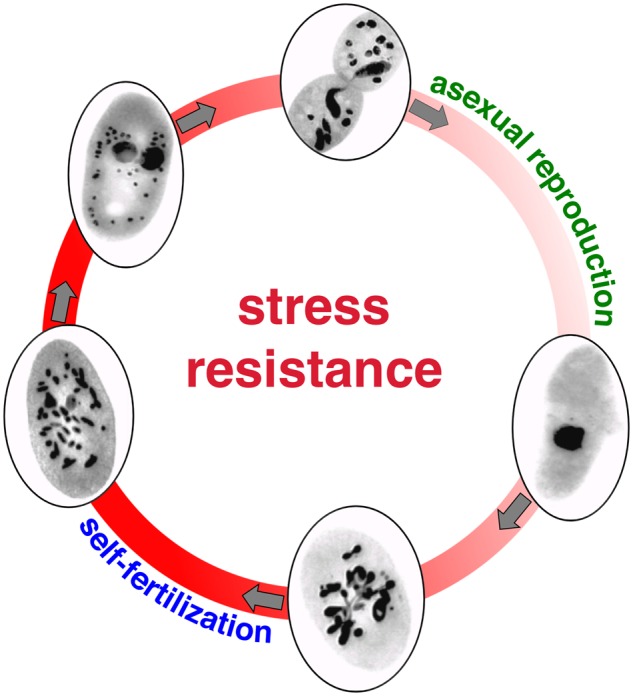
—Successive stages of self-fertilization in *Paramecium tetraurelia* and their relationship with stress resistance. Credit: Valerio Vitali.

To identify other reasons that *P. tetraurelia* may engage in sex, the researchers followed cultures of paramecia over the course of 8 days, beginning immediately after self-fertilization (day 0) and continuing past the point at which the cells again became capable of sexual reproduction (on day 6). Each day, they subjected a subset of cells to stress by heating them to a high temperature for just over a minute. Interestingly, they found that cells that had just undergone self-fertilization or that were preparing for sexual reproduction (day 0 and day 6 cells) survived the heat shock more often than those that were rapidly reproducing asexually. This survival advantage could explain why paramecia continue to engage in sex despite the fact that no new diversity is generated, and it suggests an underappreciated benefit of sex: enhanced survival in the face of stress (fig. 1).

This finding hints at a mechanistic link between sex and the stress response. The authors point out that many heat shock proteins, which are most well known for their role in protecting against stress, are also involved in cellular processes associated with reproductive development and sex. It may therefore be that the increased expression of such proteins during sexual reproduction provides added protection from stressors.

How common is this relationship between sex and stress? Although some aspects of *Paramecium* biology are unique, Catania notes that many unicellular and multicellular organisms engage in self-fertilization and, after several generations of this process, may produce offspring that are copies of their parents. In addition, many proteins involved in reproduction and the stress response are ancient and highly conserved across eukaryotes. Thus, a connection between sex and stress may be widespread, a finding that could have far-reaching implications. According to Catania, the results of their study lead to several new hypotheses about the origin and maintenance of sex: “First, the intimate association between the stress response and sex may have contributed to the persistence of sex in nature. Furthermore, these two pathways, often treated as unrelated, might in fact share a common evolutionary origin.”

Catania notes that additional studies in other organisms will be needed to test these ideas. However, there is reason to believe that their results may be generalizable to other species. “Over the years, research on *Paramecium* has yielded important insights in many areas of biology. This model has a lot more to offer despite its unusual biology, and we argue that it can be successfully used to gain new insights into many biological phenomena.” 
